# Ocular fundus changes and association with systemic conditions in systemic lupus erythematosus

**DOI:** 10.3389/fimmu.2024.1395609

**Published:** 2024-07-18

**Authors:** Lihui Meng, Yinhan Wang, Zhikun Yang, Shiqun Lin, Yuelin Wang, Huan Chen, Xinyu Zhao, Youxin Chen

**Affiliations:** ^1^ Department of Ophthalmology, Peking Union Medical College Hospital, Chinese Academy of Medical Sciences, Beijing, China; ^2^ Key Lab of Ocular Fundus Diseases, Chinese Academy of Medical Sciences, Beijing, China

**Keywords:** systemic lupus erythematosus, ocular fundus, multimodal imaging, systemic damage, kidney, central nervous system

## Abstract

Systemic lupus erythematosus (SLE) is an autoimmune disease that affects multiple organs and systems. Ocular involvement is estimated to manifest in one-third of individuals with SLE, of which lupus retinopathy and choroidopathy represent the severe subtype accompanied by vision impairment. Advancements in multimodal ophthalmic imaging have allowed ophthalmologists to reveal subclinical microvascular and structural changes in fundus of patients with SLE without ocular manifestations. Both ocular manifestations and subclinical fundus damage have been shown to correlate with SLE disease activity and, in some patients, even precede other systemic injuries as the first presentation of SLE. Moreover, ocular fundus might serve as a window into the state of systemic vasculitis in patients with SLE. Given the similarities of the anatomy, physiological and pathological processes shared among ocular fundus, and other vital organ damage in SLE, such as kidney and brain, it is assumed that ocular fundus involvement has implications in the diagnosis and evaluation of other systemic impairments. Therefore, evaluating the fundus characteristics of patients with SLE not only contributes to the early diagnosis and intervention of potential vision damage, but also holds considerate significance for the evaluation of SLE vasculitis state and prediction of other systemic injuries.

## 1 Introduction

Systemic lupus erythematosus (SLE) is an autoimmune disease involving multiple organs, with a global incidence of 5.14 per 100,000 person-years, predominantly impacting women (1). The pathology involves loss of immune tolerance against nuclear antigens, polygonal autoantibody production, and immune complex deposition, leading to multi-system damages with a relapsing clinical course (2). According to the 2019 European League Against Rheumatism/American College of Rheumatology (EULAR/ACR) criteria, patients can be classified as having SLE if they have positive ANA ≥1:80 and a score of 10 or more points (3). Global disease activity could be assessed based on Systemic Lupus Erythematosus Disease Activity Index 2000 (SLEDAI-2k), which is crucial when it comes to evaluating prognosis and treatment effects (4).

Ocular involvement is estimated to manifest in one-third of individuals with SLE (5–7), which spans various ocular structures, including the orbit, eyelids, sclera, cornea, retina, choroid, and optic nerve. The first two most common ocular manifestations of SLE are dry eye syndrome and retinopathy, the latter of which could be accompanied by severe visual impairment and even blindness (8–11). Visual impairment is closely associated with systemic disease activity and serves as an adverse prognostic marker, with a pivotal score of eight points in the SLEDAI-2k scoring system. In addition, recent studies have revealed that ocular manifestations like retinopathy and choroidopathy are correlated to specific systemic manifestations like lupus nephritis (LN) (12–14) or central nervous system (CNS) (10, 15) disease, and even subclinical alterations in retina or choroid (16–19) may harbor equivalent clinical implications.

This review aims to focus on ocular fundus changes, specifically within the retina and choroid among individuals with SLE. We endeavor to provide a concise overview of both overt manifestations and subtle structural changes, which could aid in the early detection of SLE, indicate other organ involvement, and serve as potential biomarkers of disease activity. With the aspiration to provide a comprehensive summary for individuals encountering fundus complications in SLE, our goal is to narrow the divide between ophthalmology and immunology, particularly in the context of autoimmune eye disease.

## 2 Ocular fundus manifestations in systemic lupus erythematosus

**Figure 1:**
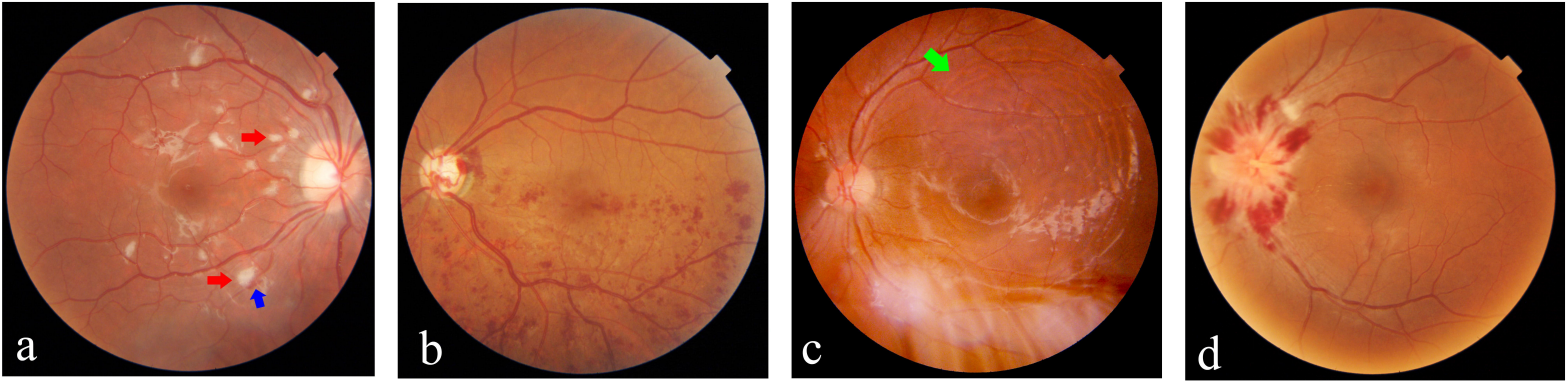
Ocular fundus lesions of patients with systemic lupus erythematosus (SLE). **(A)** Retinal microangiopathy with cotton-wool spots (red arrows) and small flame-shaped hemorrhage (blue arrow). **(B)** Branch retinal vein occlusion with tortuous and dilated veins, as well as notable hemorrhage. **(C)** Lupus choroidopathy with serous retinal detachment and subretinal fluid (green arrow). **(D)** Optic disc edema and obvious hemorrhage.

Ocular fundus damage, including lupus retinopathy (LR) and lupus choroidopathy (LC), represents the severe type of ocular manifestation in SLE, typically accompanied by significant visual impairment. Mild retinopathy could be asymptomatic while severe LR primarily presents with visual deterioration, distortions, and visual field defects. LC is commonly associated with LR, with less significant deterioration in vision acuity (20, 21). **Figure 1** provides an overview of the characteristic manifestations of retinal, choroidal, and optic disc lesions associated with SLE.

### 2.1 Retinal involvement

LR, the second most common ocular manifestation of SLE, is frequently associated with visual loss (8–11).The primary mechanisms contributing to SLE retinopathy include immune-complex-mediated microangiopathic vasculopathy, micro-thrombosis associated with endothelitis due to complement system activation or the presence of antiphospholipid (aPL) antibodies, and secondary hypertension resulting from kidney involvement and activation of the renin–angiotensin–aldosterone system (RAAS) (22).

**Figure 2:**
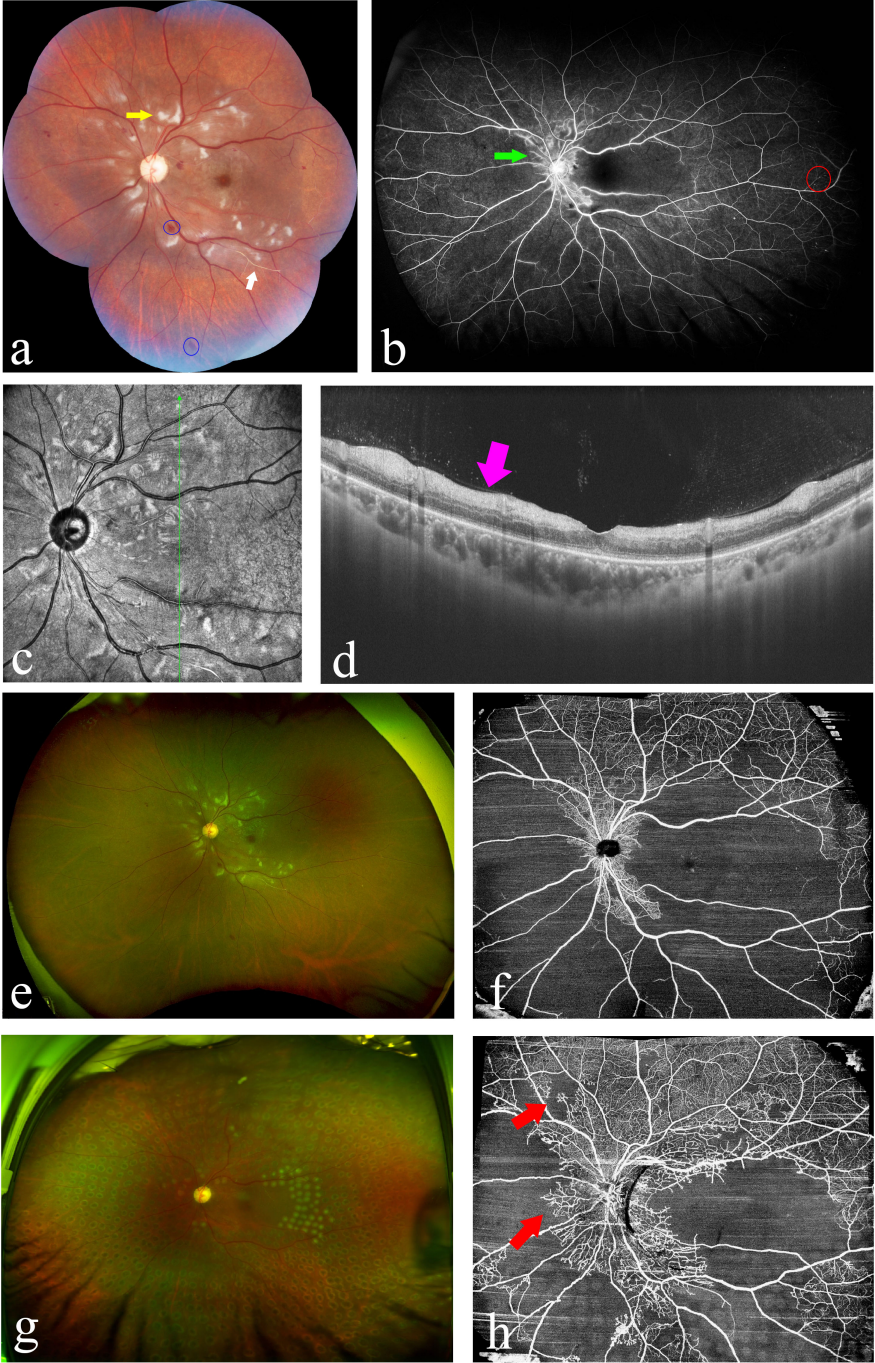
Multimodal imaging in a patient with lupus retinopathy [**(A–F)**: images were taken at the first presentation; **(G, H)**: images were taken after treatment]. **(A)** Wide-field image by montage with the traditional fundus camera demonstrated the multiple clustered cotton-wool spots (yellow arrow) and hemorrhage dots (blue circles), as well as white vascular sheathing (white arrow) (Zeiss VISUCAM 500, Carl Zeiss Meditec AG, Jena, Germany). **(B)** Wide-field fundus fluorescein angiography revealed marked area of capillary non-perfusion and vaso-occlusions, as well as vascular leakage (green arrow) and hyper-fluorescent foci (red circle) (Optos^®^ California device). **(C, D)** Optical coherence tomography (OCT) showed the irregular retinal structure and notably edema of the retinal nerve fiber layer (violet arrow). **(E)** Imaging the ocular fundus with the non-contact ultra-wide-field pseudocolor fundus camera, enabling capturing the retinal images up to 200°C (Optos^®^ California device). **(F)** OCT-angiography (OCTA) revealed significant flow void, which corresponds to the area of non-perfusion area. **(G)** Ultra-wide-field pseudocolor fundus photograph showed the stable condition of ocular fundus after laser photocoagulation (Daytona, Optos PLC, Dunfermline, United Kingdom). **(H)** OCTA detected the areas of angiogenesis and recanalization (red arrows) 10 months after the first presentation and 6 months after the laser treatment (OCT and OCTA images were taken using VG200, Svision Imaging, Ltd, Luoyang, China).

Retinal damage in SLE can be classified into three types, with microangiopathy being the most common and mildest form, vaso-occlusion representing the most severe type, and vasculitis presenting as an earlier stage of vaso-occlusion. Retinal microangiopathy is attributed to focal retinal ischemia secondary to immune complex deposition, which typically could occur covertly without any ocular symptoms, primarily characterized by cotton-wool spots and minimal retinal hemorrhage. Retinal vasculitis is characterized by acute onset and the formation of perivascular white sheathing, which is rarely seen, but once it happens, it could lead to severe visual impairment with secondary retinal ischemia, hemorrhage, and edema. A minority of LR patients may experience central or branch retinal artery/vein occlusion, with a poor visual prognosis. Furthermore, the subsequent development of neovascularization in some cases may lead to subretinal hemorrhage or even retinal detachment. Development and resolution of LR lesions are believed to parallel the course of systemic disease (23), as retinal blood vessels might serve as a window for the visualization of systemic vascular inflammation status. **Figure 2** presents the multimodal ophthalmologic imaging features of LR.

Apart from retinopathy related to autoimmune reactions, drug-associated toxicity, particularly from the antimalarial agent hydroxychloroquine (HCQ), is another major concern for many patients and clinicians. The prevalence of HCQ retinopathy is approximately 1.6%–6.8% for HCQ use of less than 5 years (24, 25), but it can rise to 7.5%–10% with prolonged treatment (26, 27). The pathogenesis of HCQ-induced retinal toxicity is poorly understood (28). *In vitro* studies suggest that HCQ accumulates in melanin-containing cells, leading to lysosomal alkalinization and inhibition of autophagy (29). This process may contribute to the accumulation of lipofuscin, potentially playing a role in its retinal toxicity.

Individuals with HCQ retinopathy might not manifest symptoms initially. However, they can advance to exhibit thinning of the outer retina, photoreceptor, and retinal pigment epithelium (RPE) in the parafoveal or pericentral region, which could result in the bull’s-eye pattern in the late stage (30). The area of functional disturbance expands as drug exposure continues with progressive and irreversible loss of vision, and the maculopathy can encroach on the foveal center with eventual blindness (31). Early detection of HCQ retinopathy through retinal screening is essential and continues to be a challenge. A nationwide interrupted time-series analysis conducted in Korea revealed a rise in the annual screening test implementation rate among new HCQ users, increasing from 3.5% in 2006 to 22.5% in 2019 (32). Despite this improvement, there is still a need to raise awareness on retinal screening in clinical settings.

### 2.2 Choroidal involvement

LC is rarely seen in clinics compared with LR, and no more than 60 cases have been reported to date (13, 33–35). It commonly manifests with bilateral serous or exudative retinal detachment, an increase in choroidal thickness, and the presence of subretinal deposits corresponding to immune complexes (36, 37). Unilateral or bilateral blurred vision is the most common presenting sign with an acceptable visual acuity of 20/40 or better in approximately two-thirds of the patients (13). The exact pathology of LC remains uncertain, but it is widely acknowledged to involve various factors. These factors may include choroidal dysfunction and damage to RPE due to immune complex deposition and thrombosis development in the choroidal vasculature. This cascade of events may lead to subsequent retinal pigment epitheliopathy and leakage of serous fluid (13, 38).

The choroid can also be affected by the adverse effects of systemic management in SLE. Central serous chorioretinopathy (CSC) may occur after the use of glucocorticoids, which is characterized by the acute onset of subretinal fluid and serous retinal detachment, with limited RPE alterations that tend to resolve in 3–6 months (39). It usually involves the unilateral eyes with complaints of blurred vision and visual distortion. Clinicians may have to discontinue steroid therapy in these cases to prevent further impact on choroidal vasculature (40). Therefore, it must be differentially diagnosed with LC because of opposite therapeutic strategies.

## 3 Subclinical changes of ocular fundus in systemic lupus erythematosus

Even in patients with SLE without ocular manifestations, numerous investigations have detected subclinical ocular alterations through fundus screening. These important discoveries benefit from advancements in multimodal imaging techniques such as optical coherence tomography (OCT) and optical coherence tomography angiography (OCTA). OCT is a quick, repeatable, and cost-effective examination and provides highly detailed images of the retina and choroid structure in distinct layers, as shown in **Supplementary Figure 1**. The emerging technique OCTA could provide additional information relying instead on the movement of particles like red blood cells to capture images and quantitative measurements of the retinal and choroidal vasculature, even in the different sub-layers of the retina, as shown in **Supplementary Figure 2**. These imaging techniques have allowed ophthalmologists to evaluate the microvascular and structural changes of ocular fundus in a more comprehensive and non-invasive way. Moreover, traditional invasive fundus imaging techniques like fluorescein angiography (FFA) and indocyanine green angiography (ICG-A) that require the intravenous injection of fluorescein/indocyanine green (ICG) dye give information on retinal and choroidal vasculature.

**Figure 3:**
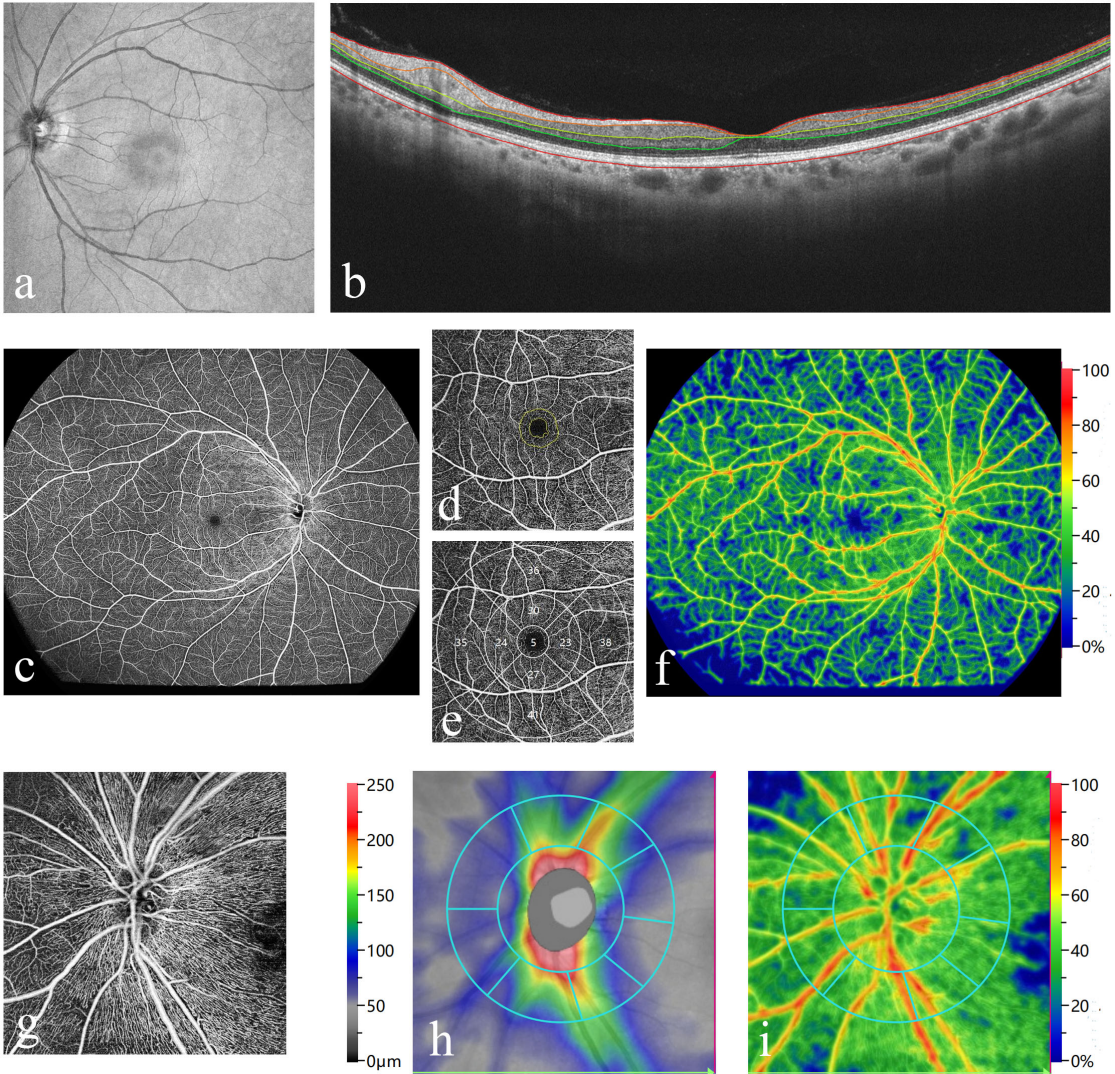
Measurement of retinal thickness with swept source optical coherence tomography (OCT) and vasculature parameters with OCT-angiography (OCTA) to detect the subclinical changes in a patient with SLE (Images were taken using BM400K BMizar, TowardPi Medical Technology Co., Ltd, Beijing, China). **(A, B)** Infrared image coupled OCT with automated segmentation of retinal layers [line red: internal limiting membrane (ILM); line orange: retinal nerve fiber layer (RNFL); line yellow: inner nuclear layer; line green: outer nuclear layer; dark red: Bruch’s membrane]. **(C)** A wide-field OCTA image of retinal superficial capillary plexus from ILM to the inner plexiform layer. **(D)** Measurements of fovea avascular zone (FAZ) parameters, including area, acircularity, and FAZ vessel density—300 μm. **(E)** Vessel densities of four quadrants of the parafoveal and perifoveal area. **(F)** The vessel density map of the wide-field OCTA image. **(G)** An image of radial peripapillary RNFL capillary plexus. **(H)** Peripapillary RNFL thickness map. **(I)** Peripapillary RNFL vessel density map.

Individuals diagnosed with SLE exhibit alterations in the vascular architecture and layer structure of the fundus (41). The early structural or vasculature changes of the retina and choroid in patients with SLE are summarized in **Table 1**. **Figure 3** gives an overview of how to detect the subclinical changes in retinal and choroidal structure and microvasculature with swept-source OCT and OCTA in patients with SLE.

**Table 1:** Subclinical changes of ocular fundus in patients with SLE by multimodal ophthalmic imaging.

Imaging	Findings
FFA/ICGA	Venular occlusion and optic nerve leakage;
	Alternating hyper-fluorescence and hypo-fluorescence outside the arcades as well as peripapillary areas and capillary dropout;
	Drusen-like deposits indicating choroidal changes.
OCT	Reduced central macular retinal or sub-layer thickness;
	Choroidal thickness changes in sub-foveal and peri-foveal area.
OCTA	Reduced retinal microvascular density and perfusion of the superficial and deep retinal capillary plexuses referred to the whole en face, foveal, and parafoveal zone;
	Reduced FAZ circularity;
	Enlargement of FAZ.

FAZ, fovea avascular zone; FFA, fluorescein angiography, ICGA, indocyanine green angiography; OCT, optical coherence tomography; OCTA, optical coherence tomography angiography; SLE, systemic lupus erythematosus.

### 3.1 Retinal subclinical changes

The retina is nourished by three vascular sources: central retinal blood vessels supply the inner layers, choroidal vessels (part of the ciliary vascular system) supply the outer layers, and the macular region is supported by choroidal capillaries. Systemic vasculitis associated with SLE or its treatment may affect both retinal and choroidal vasculature, thereby impacting the supply of oxygen and nutrients to neighboring cells and further resulting in structural damage, evident through alterations in layer thickness, vessel density, and lumen volume (42). Multimodal ocular fundus imaging techniques enable the visualization and quantification of these subtle structural and vascular changes before they manifest as vision-related symptoms over prolonged exposure.

Decreased macular vessel density has been demonstrated by many cohort studies in all layers of the retina, including the superficial capillary plexus (SCP), intermediate capillary plexus (ICP), and deep capillary plexus (DCP) in patients with SLE without ocular involvement (17, 42–45). A meta-analysis incorporating 1,246 eyes from 1,013 patients has reported that patients with SLE without ocular fundus damage presented a reduction of macular vessel density of both SCP and DCP in all zones (whole scan, fovea, parafovea, and perifovea) and of foveal density compared with healthy controls, while no differences were found in terms of foveal avascular zone parameters (23). It is highly suspicious that SCP and DCP impairment could lead to insufficient oxygen and nutrient supply to the inner retina, causing changes in the retinal structure and contributing to the development of LR. On the other hand, deep retinal capillary plexus impairment could be considered as an early disease activity biomarker, which may cause gradual obstruction followed by hemodynamic dysfunction and photoreceptor integrity loss (43). Guo et al. have revealed that the foveal vessel density further decreased in LR patients compared to SLE without ocular involvement. Interestingly, among subjects positive for anti-dsDNA antibodies and with increased disease activity, a significant decrease in foveal vessel density was noted (41).

Besides microvascular alterations, OCT has revealed structural changes in patients with SLE without ocular involvement. RNFL (axons), ganglion cell layer (cell bodies), and inner plexiform layer (dendrites) together comprise the ganglion cell complex, which is responsible for transmitting visual information from the photoreceptors to the optic nerve. Subclinical retinal changes of reduced photoreceptor layer, peripheral retinal nerve fiber layer (RNFL) (19), ganglion cell layer, and inner plexiform layer thickness (46) were observed in patients with SLE, thus presenting early signs of retinal neurodegeneration.

FFA can also detect retinal changes in patients with SLE without ocular manifestations, which may present as venous congestion and tortuosity, RPE degenerative changes in the form of alternating hyperfluorescence and hypofluorescence, or diffuse mottling (47, 48). However, this procedure has two major pitfalls: invasiveness and the lack of quantitative function, precluding its widespread application in screening patients with SLE for subclinical ocular involvement.

### 3.2 Choroidal subclinical changes

The choroid is immediately adjacent to the outer retina and organized as a dense network of small blood vessels with a large surface area, facilitating the exchange of oxygen and nutrients to the outer layers of the retina. In addition to the retina, choroidal changes have also been proven by the decreased macular thickness in patients with juvenile SLE without ocular manifestations compared to age- and sex-matched controls (49). Thinning of the choroidal structure is believed to correlate with hypoperfusion of the choroidal circulation, possibly attributed to vasculitis and chronic ischemia. Given that the vascular and connective tissue of the choroid develop and expand progressively, the choroid typically thickens with age during childhood. It is also hypothesized that the diagnosis of juvenile SLE might be delayed and the exact disease duration might be underestimated.

However, in contrast to the patients with SLE with no ocular disease, those with retinopathy exhibit increased choroidal thickness and increased luminal, stromal, and total choroidal area (41). This possibly arises from systemic factors such as SLE duration, severity, and activity, which may exert a stronger influence on choroidal vasculature than the local ocular fundus microenvironment. Hence, caution should be warranted during fundus imaging cohort studies to minimize potential bias originating from systemic factors.

FFA and ICG-A have also proven to be effective in detecting choroidal abnormalities resembling drusen-like deposits (DLDs) in patients with SLE without ocular symptoms. DLDs exhibit early pinpoint hyperfluorescence that remained unchanged during FFA and well-defined hyperfluorescent spots from the intermediate to the late phase of ICG-A. These spots are proposed to be immune complex and complement deposition between Bruch’s membrane and RPE (50). Furthermore, in eyes with DLDs, reductions in the choroidal vascular index (CVI, the proportion of vascular areas to the total choroidal area, representing the percentage of medium- and large-sized choroidal vessels in the choroid) and the photoreceptor cell layer thickness have been observed (51), likely attributable to immune infiltration causing stromal area expansion and vascular lumen occlusion by immunocomplexes. This reduction in CVI may lead to ischemia and influence the nutrient and oxygen supply of retinal segments, leading to further atrophy and decrease in photoreceptor layer thickness. Therefore, it is suggested that DLDs, as a possible indicator of choroidopathy, could be a sign of advancing disease.

## 4 Ocular fundus imaging assisted early diagnosis of SLE and disease activity evaluation

Actually, active inflammation in the retina and choroid can simulate systemic vasculitis found in other organs due to a shared pathological basis (20). The observations in the posterior segment, especially retinal manifestations, frequently reflect the increased disease activity (assessed by SLEDAI-2k) and indicate inadequate management of systemic inflammation (52). This suggests that fundus imaging techniques could potentially identify biomarkers for microvascular changes in patients with SLE, offering a more effective approach to studying the disease state and predicting further systemic damage.

### 4.1 Early diagnosis of SLE

Ocular symptoms could be detected sometimes at the onset of SLE or revealed at initial diagnosis, usually recognized as pain (often accompanied by visible inflammation or redness, indicating significant external/anterior segment disease) or vision disturbances (blurring, distortion, and double vision, indicating posterior segment/neuro-ophthalmic disease) (6, 53). Retinal and choroidal vasculopathy in SLE may reflect the potential endothelial damage caused by the background dysregulated immune system (22, 54, 55). When an ophthalmologist detects severe ocular complications like retinal vasculitis, scleritis, ulcerative keratitis, or corneal perforation, particularly in cases of bilateral ocular involvement, autoimmune diseases such as SLE should be considered among the differential diagnoses (20). Several studies have evaluated the diagnostic accuracy of certain subclinical changes such as retinal vessel densities or thickness. Subasi et al. found that the DCP-vessel density in OCTA had a good sensitivity and specificity when discriminating between SLE and controls (AUC value = 0.671) (17). Liu et al. found that in the outer temporal region, the AUC value of the inner retinal thickness for diagnosing SLE was 0.805, while the full retinal thickness was 0.828 (56). Therefore, utilizing these subclinical changes of retina might be a new approach to diagnose SLE.

### 4.2 Association between ocular fundus changes with SLE disease activity

An increasing number of evidence suggest a correlation between ocular fundus involvement and systemic disease activity in SLE. LR is a significant component, comprising eight points in the SLEDAI-2k scoring system. Patients with LR had a significantly higher SLEDAI-2K score (52). LC also indicates active lupus (57). Given that high disease activity often leads to severe symptoms and poorer prognosis, ocular fundus involvement warrants significant attention in patients with SLE.

Moreover, subclinical retinal or choroidal changes have been linked to disease activity assessed by SLEDAI-2k. Ermurat et al. observed negative correlations between retinal vessel densities of SCP and DCP and disease activity (58). Lee et al. reported increased choroidal thickness in patients with SLE with high disease activity (59). However, a recent systematic review highlighted insufficient and variable studies to estimate the incidence of ophthalmic complications associated with disease activity (60). Some studies even yielded contradictory results regarding choroidal thickness changes in SLE (49, 61). This inconsistency underscores the dynamic nature of retinal and choroidal alterations, possibly influenced by disease duration, severity, or inflammatory response (49, 62). Hence, larger multicenter cohort studies are warranted to validate associations between ocular manifestations and systemic disease activity in SLE, while mitigating bias from other systemic factors. Future prospects might involve incorporation of specific fundus lesions or imaging features into the new systemic disease activity scoring system.

### 4.3 Other systemic indicators

Besides disease activity, ocular parameters may correlate with disease duration, inflammation markers, or autoantibody profiles. Pelegrin et al. found that OCTA parameters, including vessel density, vessel perfusion and fovea avascular zone (FAZ) circularity, and macular thickness, were decreased in patients with longer disease duration (63). The FAZ refers to a capillary-free area in the macula characterized by the highest density of cone photoreceptors. Its circularity reflects its geometric configuration, and alterations therein may impact fine vision. RNFL is also a critical layer of the retina, consisting of axons of ganglion cells responsible for transmitting visual information from the photoreceptors to the optic nerve. Thinning of the RNFL may indicate nerve damage or degeneration, while thickening may suggest inflammation or edema. Patients with SLE with reduced RNFL thickness were associated with the presence of aPL antibodies or secondary aPL syndrome (APS) (63). Choroidal thickness may also be associated with cytokine profiles. Temporal and subfoveal choroidal thickness are negatively correlated with interleukin (IL)-6 and IL-10 (49). However, as stated above, the conclusions need to be confirmed in larger cohort studies.

## 5 Associations between ocular fundus and other systemic involvement

According to statistics, organs that are most commonly involved in SLE include the joints, skin, kidneys, hematopoietic system, and CNS, among which kidney involvement has a prevalence of 5.4%–53.5%, and CNS involvement is estimated to be 10.3%–36.9% (64–67). LN and neuropsychiatric systemic lupus erythematosus (NPSLE) remain challenges for clinicians at both diagnostic and therapeutic aspects and continue to provide significant morbidity and mortality (68). The diagnosis and treatment of LN and NPSLE depend largely on invasive manipulations such as renal biopsy or lumbar punctuation.

**Figure 4:**
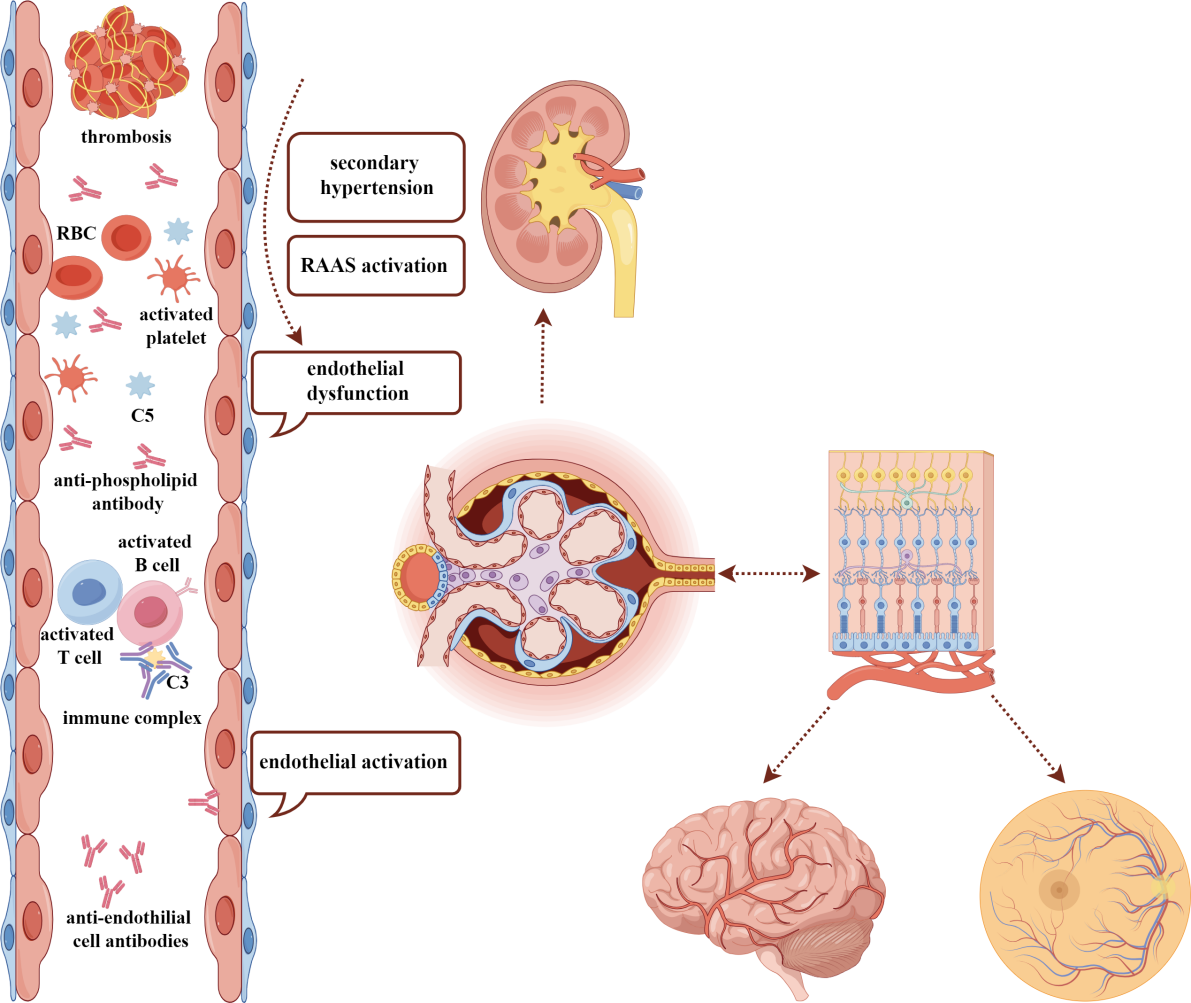
The similar pathogenesis among ocular fundus, kidney, and brain damage in SLE. The common pathogenic factors, including immune-complex deposition, endothelial dysfunction, activation of RAAS, secondary hypertension, and existence of antiphospholipid antibodies may contribute to the damage of kidney, ocular fundus, and brain, which have a similar anatomy. SLE, systemic lupus erythematosus; RAAS, renin–angiotensin–aldosterone system; RBC, red blood cell.

The eye is considered a window to the kidney and brain. Several structural, developmental, and organizational similarities, including Bruch’s membrane and glomerular basement membrane, and chorioretinal and renal microcirculation, support the hypothesis that ocular changes might present as a reflection of the renal disease. The eye is an anatomical extension of the brain where multiple parallels can be drawn between their neurons, vasculature, and immune response (69). In SLE, immune-complex-mediated microvascular damage, secondary hypertension consequent to kidney involvement with activation of the RAAS, and the micro-thrombosis associated with endothelitis or aPL antibodies are the main mechanisms leading to ocular fundus lesions (22), which shares similarity with the pathogenesis of LN and NPSLE (**Figure 4**) (70, 71). In addition, endothelial dysfunction is an important feature and facilitating factor at the early stage of SLE (72). These associations lead to the hypothesis that the eye could serve as a window to detect other systemic damage. Furthermore, it offers a great advantage and convenience in terms of investigating the intraocular vasculature with multimodal ophthalmic imaging. Early detection of fundus lesions might call attention to early renal function and brain magnetic resonance imaging (MRI) examinations and enable early detection of vital organ involvement, preventing severe systemic response.

### 5.1 Ocular changes associated with lupus nephritis

LN is considered a robust predictor of morbidity and mortality in SLE, leading to end-stage renal disease in 5.4%–53.5% of patients (64–67). While renal biopsy remains the gold standard for LN diagnosis, its invasiveness limits frequent and serial applications. Clinical biomarkers, such as urine protein and serum creatinine, could be inconsistent with histologic conditions in approximately 30% of cases (73). Therefore, an accurate and noninvasive method to assess renal disease activity and response to therapeutic interventions is urgently needed. Prior research has identified various non-invasive techniques for predicting LN, such as flow-mediated dilation of the brachial artery. Given the documented associations between ocular changes and LN, we believe that these changes could also serve as potential noninvasive screening methods for LN (72).

Firstly, the common coexistence of LN and retinopathy suggests a potential association between them. The prevalence of LN was significantly higher in those with retinopathy (12), and increased serum creatinine or proteinuria was found in patients with LR (74, 75). Cluster analysis revealed the co-appearance of ocular and renal damages (76). Two pertinent studies indicated that patients with both renal and ocular damage were more likely to be categorized into the same cluster with the highest risk of mortality and the highest disease activity (77, 78). In addition, in patients with SLE, researchers noted significant associations between both renal and ocular damage and thrombocytopenia in patients with SLE (79). Furthermore, associations between LN and subclinical ocular involvement have been evidenced by reduced retinal vessel density in patients with SLE with renal involvement, as uncovered by OCTA (80).

Secondly, associations between choroidal vascular alterations and kidney involvement have primarily been identified through abnormal fluorescence in ICG-A and choroid thickness by OCT. Baglio et al. observed DLDs in all patients with LN using ICG-A. They suggested that ICG-A abnormal findings could aid in determining the necessity of renal biopsy, particularly in cases where histological lesions might be present despite normal urinary sediment and/or proteinuria (50). In addition, Gharbiya et al. investigated ICG-A findings in patients with LN, revealing two patterns: early-phase focal, transient hypofluorescent areas possibly due to choroidal filling delay, and late-phase spots or choroidal hyperfluorescence indicating localized abnormal binding of the ICG molecule, likely representing immune deposits (81). These findings imply a close association between choroidal and renal involvement.

As for choroid thickness, Braga et al. found that the choroid in the LN group was significantly thicker than that in the SLE control and healthy control groups (16). Hassan et al. also found that the subfoveal choroid was significantly thicker among the patients with LN as compared to the non-nephritic subgroup. Furthermore, they conducted molecular investigations and found that miRNA146 had a significant positive correlation with patients with LN. Thus, the authors proposed that the subfoveal choroid thickening, in combination with increased miRNA 146 levels, could be considered as a potential biomarker in LN (82).

### 5.2 Ocular changes associated with central nervous system involvement

CNS involvement has been reported to occur in 10.3%–36.9% of patients with SLE (64–67), a wide range of whose prevalence is mainly due to a multitude of manifestations recognized as NPSLE (83). Diagnosis of NPSLE remains a great challenge as neurologic conditions might arise from multiple causes like post-steroid adverse effects or attribution to SLE, and subclinical involvement is lacking available biomarkers. Therefore, NPSLE is often detected and diagnosed at its late stages with poor prognosis and often associated with cognitive dysfunction.

Non-invasive imaging methods for CNS evaluation in SLE include MRI, revealing atrophy in the frontal and temporal gray and white matter as a hallmark. However, up to 50% of patients with NPSLE have normal examination results and non-NPSLE patients can also be captured with abnormal brain scans, indicating that the sensitivity and specificity of MRI are insufficient to diagnose CNS involvement (70). In addition, non-invasive approaches to assessing vasculopathy of CNS are limited when evaluating the cerebral microvasculature. Therefore, biomarkers or invasive screening methods for early diagnosis are in urgent need.

Visual system involvement is highly prominent in NPSLE (73% of cases), especially retinal involvement (84, 85). OCT and OCTA can evaluate the retinal vasculature and structural changes, with strong correlations to those in the brain. In patients with neurological disorders including Alzheimer’s disease and stroke, it was demonstrated that RNFL thickness was positively associated with brain volume, ganglion cell layer thickness was positively associated with regional cerebral blood flow, and reduction of the vessel density and perfusion in the superficial capillary plexus correlated with expansion of the inferolateral ventricle, indicating that OCT/OCTA might be employed as useful non-invasive and convenient tools for the detection of brain neurodegeneration (86–88). However, there are limited publications on the use of fundus imaging in assessing NPSLE. Previous research has shown that patients with SLE presented early signs of neurodegeneration, evidenced by a progressive reduction in peripheral RNFL and photoreceptor layer thickness as assessed by OCT (19, 89, 90), while there is a lack of association between these fundus neuro-structural changes and secondary neuropsychiatric symptoms in longitudinal studies. A case report has observed simultaneous vasculopathy on the small retinal and large cerebral vessels by OCTA and magnetic resonance angiography (MRA) in NPSLE (85), while studies with a larger sample size are needed to provide evidence that fundus vessels may reveal intracranial vascular involvement.

### 5.3 Ocular changes associated with the involvement of other systems

The involvement of other systems reported to be associated with ocular changes includes hematological disturbance and systemic serositis. Gao et al. demonstrated that leukocytopenia was an independent risk factor for retinal vasculopathy in SLE (91). Ishida et al. reported that multiple ocular effusions often accompanied the signs of systemic serositis including pleuritis, pericarditis, and peritonitis. Conjunctival chemosis, annular ciliochoroidal detachments, retinal edema, and subfoveal serous retinal detachment were relieved when the serositis was resolved (92). This may prompt doctors to think from the perspective of anatomy and pathological anatomy when managing patients of systemic diseases, which are likely to involve organs and systems with a common anatomical basis. Systemic monitoring and detecting early symptoms may help prevent serious outcomes.

## 6 Future perspectives and conclusion

In this review, we have focused on ocular fundus features, which can potentially help with early diagnosis, the prediction of systemic disease activity, and the involvement of other systems in SLE. While accumulating evidence highlights the importance of early fundus screening for detecting subclinical ocular changes, it is essential to acknowledge the limitations of existing publications, which mainly comprise cohort studies with small sample sizes and case reports, leading to some contradictory conclusions. In addition, by reviewing the current literature, we observed significant heterogeneity in the clinical and subclinical manifestations of ocular involvement in SLE. Higher-quality studies with larger sample sizes are imperative to provide proof of concept, whether in all patients with SLE or in a small subgroup, that subclinical structural changes precede ocular manifestations and even involvement of other systems, and associate with systemic disease activity. For those identified with early changes in fundus imaging features, the integration of advanced technologies, such as machine learning or deep learning, might be employed to identify common traits and facilitate early diagnosis of SLE.

For patients with LR or LC already, systemic treatment and local therapy should be intensified. Close monitoring is also recommended for patients with subclinical retinal and choroidal changes, addressing both ocular and systemic conditions in SLE. Given that the retinal and choroidal structure and microvasculature vary with disease activity and duration in SLE, dynamic analysis of these parameters is crucial to explore the predictive value of ocular fundus changes in systemic organ damage. General practitioners or rheumatologists could initiate screening for ocular lesions in rheumatic diseases like SLE using color fundus photography and OCT. However, early ophthalmic consultation is strongly recommended.

In addition to heightened vigilance for ocular symptoms and structural changes in patients with SLE, efforts should be directed toward characterizing individuals who might exhibit evidence of ocular involvement. Though it would be ideal for each SLE patient to undergo ophthalmic clinical examinations, we recognize that medical resources and treatment expenses remain a challenge for some patients. Therefore, further studies are warranted to explore the relationship between ocular symptoms, fundus imaging changes, and specific peripheral biomarkers. This exploration could guide rheumatologists in identifying indicators that may prompt timely fundus screening in patients with SLE (86).

Transitioning from bedside to bench, there is a critical need to understand how the blood–ocular barrier is broken through and an excessive immune response explodes out of control in the eyes, which, under normal circumstances, sustains an immunosuppressive and tolerant environment to prevent eyes from incurring inflammatory damage. Moreover, investigating the similarities and differences in immunological mechanisms between local immune responses attacking fundus tissue and systemic reactions affecting other organs is crucial, which might provide new treatment targets for SLE.

In conclusion, it is recommended for patients with SLE to receive ophthalmologic screening at the time of diagnosis, with regular monitoring during the clinical course particularly for those with severe disease or heightened disease activity. We emphasize the importance of paying greater attention to ocular manifestations in patients with SLE, which might be a danger signal for rapid deterioration and serious outcomes, not only limited to ocular lesions but also affecting other vital organ systems.

## 7 Literature search

Embase, Medline, PubMed, and Web of Science databases were searched for all relevant studies published from inception to February 2024. The search terms used were as follows: “Systemic lupus erythematosus”, “Eye”, “Ocular fundus”, “Retina”, “Choroid”, “Multimodal imaging”, “Fluorescein angiography”, “Indocyanine green angiography”, “Optical coherence tomography”, “Optical coherence tomography angiography”, “Nephritis”, and “Central nervous system”. All relevant publications were limited to the English language. Prospective and retrospective studies involving the ocular fundus damage in SLE, retinal changes, choroidal changes, and systemic conditions were included. Moreover, we included case reports and letters only if they contributed new information and perspectives about the detection and significance of ocular fundus damage in SLE. Publications in other languages, repetitive studies, and animal or laboratory experimental studies were excluded.

## Author contributions

LM: Writing – review & editing, Conceptualization, Investigation, Visualization, Writing – original draft. YHW: Conceptualization, Funding acquisition, Investigation, Visualization, Writing – original draft, Writing – review & editing. ZY: Writing – review & editing. SL: Writing – review & editing. YLW: Writing – review & editing. HC: Project administration, Resources, Supervision, Writing – review & editing. XZ: Project administration, Resources, Supervision, Writing – review & editing. YC: Funding acquisition, Project administration, Resources, Supervision, Writing – review & editing.
