# Supplementary material for: Ocular fundus changes and association with systemic conditions in systemic lupus erythematosus
Source: Front Immunol. 2024 Jul 18;15:1395609. doi: 10.3389/fimmu.2024.1395609 (PMC11291259; doi:10.3389/fimmu.2024.1395609)
Supplement: Supplementary file 1 [file DataSheet_1.docx]

Supplementary Material

Ocular fundus changes and association with systemic conditions in systemic lupus erythematosus

**Lihui Meng^1,2†^, Yinhan Wang^1,2†^, Zhikun Yang^1,2^, Shiqun Lin^1,2^, Yuelin Wang^1,2^,** **Huan Chen^1,2*^, Xinyu Zhao^1,2*^, Youxin Chen^1,2*^**

^1^ Department of Ophthalmology, Peking Union Medical College Hospital, Chinese Academy of Medical Sciences, Beijing 100730, China

^2^ Key Lab of Ocular Fundus Diseases, Chinese Academy of Medical Sciences, Beijing 100730, China

Lihui Meng^1,2†^, Yinhan Wang^1,2†^ These authors contributed equally to this work and share first authorship.

Huan Chen^1,2^**^*^**, Xinyu Zhao^1,2^**^*^**, Youxin Chen^1,2^**^*^** These authors contributed equally to this work and share last authorship.

*** Correspondence:**You-xin Chen MD, PhD, Professor
chenyx@pumch.cn

Xinyu Zhao, MD
zhaoxinyu@pumch.cn

Huan Chen, MD
[chenhuan1@pumch.cn](mailto:chenhuan1@pumch.cn)

**Supplementary Figures**


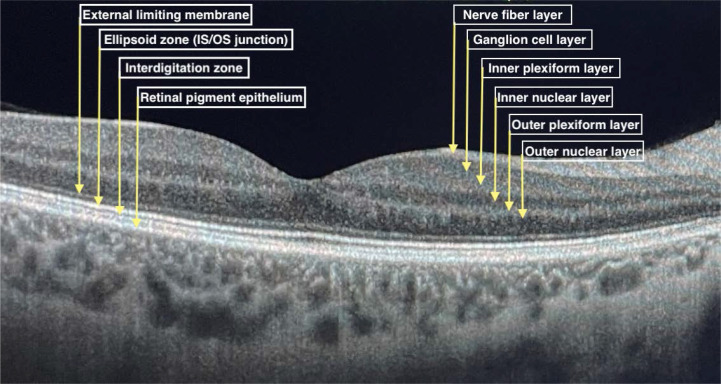


**Supplementary Figure 1.** Optical coherence tomography scan of a healthy retina and segmentation of individual layers.(1) Copyright: © 2023 Narodowy Instytut Geriatrii, Reumatologii i Rehabilitacji w Warszawie, CC BY-NC-SA 4.0 License


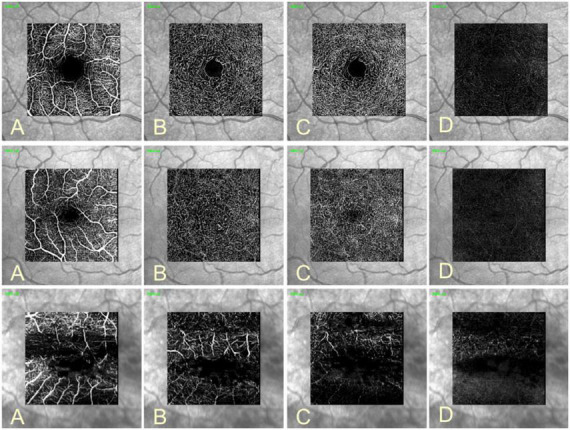


**Supplementary Figure 2.** Presentation of optical coherence tomography angiography images of a healthy right eye and quantitative parameters. The microvasculature of the superficial (A), the intermediate (B), the deep vascular plexus (C) and the avascular plexus (D) are shown. (2)Copyright: © 2023 Böhm, Pfeiffer, Wagner and Gericke.

**Reference:**

1. Mimier-Janczak MK, Kaczmarek D, Proc K, Misiuk-Hojło M, Kaczmarek R. Subclinical retinopathy in systemic lupus erythematosus patients - optical coherence tomography study. Reumatologia. 2023;61(3):161-8.

2. Böhm EW, Pfeiffer N, Wagner FM, Gericke A. Methods to measure blood flow and vascular reactivity in the retina. Front Med (Lausanne). 2022;9:1069449.
